# Histological Evidence of the Antifibrotic Effect of Polyphenolic Extracts from the Berries *Vaccinium uliginosum*, *Vaccinium oxycoccos*, and *Vaccinium vitis-idaea* in a Model of Pulmonary Fibrosis

**DOI:** 10.3390/biomedicines14081816

**Published:** 2026-08-12

**Authors:** Zarina Shulgau, Yevgeniy Kamyshanskiy, Shynggys Sergazy, Lyudmila Kovalenko, Madina Baurzhan, Sayagul Kairgeldina, Alexander Gulyayev

**Affiliations:** 1 Research Institute of Balneology and Medical Rehabilitation, Astana 010000, Kazakhstan; sergazy.shynggys@gmail.com (S.S.); madina_baurzhan@mail.ru (M.B.); sanborovoe@mail.ru (S.K.); akin@mail.ru (A.G.); 2 Department of Science Development, Astana Medical University, Astana 010000, Kazakhstan; 3 School of Medicine, Medical University Karaganda, Karaganda 100009, Kazakhstan; kamyshanskiy84@mail.ru; 4 School of Medicine, Surgut State University, Surgut 628400, Russia; kovalenko_lv@surgu.ru

**Keywords:** pulmonary fibrosis, bleomycin, blood hyperviscosity, polyphenolic extract, antifibrotic effect, hemorheological effect

## Abstract

**Background**: Pulmonary fibrosis is a chronic and progressive disease characterized by excessive deposition of extracellular matrix and scarring of lung tissue, resulting in a gradual decline in respiratory function. Despite advances in understanding its pathogenesis, effective therapeutic options remain limited. This study aimed to evaluate the antifibrotic effects of concentrated polyphenolic extracts obtained from bilberry (*Vaccinium uliginosum*), cranberry (*Vaccinium oxycoccos*), and lingonberry (*Vaccinium vitis-idaea*) in a bleomycin-induced rat model of pulmonary fibrosis. **Methods**: Pulmonary fibrosis was induced in rats through the intratracheal administration of bleomycin. Beginning seven days after fibrosis induction, concentrated polyphenolic extracts from the three Vaccinium species were administered orally for 14 consecutive days. Lung histomorphology, inflammatory cell infiltration, collagen composition, and hemorheological parameters were subsequently evaluated. **Results**: Treatment with the berry polyphenolic extracts reduced the severity of bleomycin-induced pulmonary fibrosis and decreased inflammatory cell infiltration in lung tissue. The extracts also influenced collagen remodeling, promoting a relative predominance of immature type III collagen over mature type I collagen. Furthermore, bleomycin-induced pulmonary fibrosis was accompanied by increased blood viscosity, whereas treatment with the polyphenolic extracts partially normalized the altered hemorheological parameters. **Conclusions**: Concentrated polyphenolic extracts derived from *Vaccinium* species demonstrated antifibrotic, anti-inflammatory, and hemorheological effects in experimental pulmonary fibrosis. These findings support further investigation of their underlying molecular mechanisms and potential translational applications studies.

## 1. Introduction

Pulmonary fibrosis comprises a heterogeneous group of chronic interstitial lung diseases characterized by excessive deposition of extracellular matrix, progressive destruction of normal lung architecture, and irreversible decline in respiratory function. Occupational pulmonary fibrosis is caused by long-term coal dust inhalation and associated with a spectrum of respiratory diseases collectively referred to as coal dust-associated lung disease (CMDLD) [1]. In addition to coal workers’ pneumoconiosis, miners have an increased risk of chronic airway diseases, including emphysema and chronic bronchitis. All conditions in the CMDLD spectrum can progress to coal dust-associated diffuse pulmonary fibrosis (PF) [2]. PF represents the end-stage of occupational lung diseases such as dust-associated bronchitis and anthracosis (miners’ pneumoconiosis) [3,4,5].

Despite substantial advances in understanding the molecular mechanisms underlying pulmonary fibrosis, its pathogenesis remains incompletely understood. Although currently available antifibrotic therapies can slow disease progression in selected patients, they do not reverse established fibrosis or restore normal lung architecture. The development of pulmonary fibrosis is a multifactorial process involving persistent tissue injury, chronic inflammation, dysregulated wound healing, fibroblast activation, excessive extracellular matrix deposition, and progressive scar formation [6,7]. In occupational lung diseases such as coal workers’ pneumoconiosis, these pathological processes are initiated by prolonged inhalation of toxic coal dust particles. The coexistence of chronic inflammation and fibrosis progressively impairs gas exchange, resulting in dyspnea, reduced quality of life, respiratory failure, and ultimately death [8,9,10]. Because pulmonary fibrosis develops gradually over time, therapeutic intervention before the establishment of irreversible scar tissue remains a rational strategy for limiting disease progression [11].

Because pulmonary fibrosis involves multiple interconnected pathogenic pathways, effective therapeutic approaches are likely to require modulation of several biological processes rather than a single molecular target. In this context, polyphenols have attracted considerable interest because of their pleiotropic biological activities and their ability to influence multiple cellular pathways [12,13,14]. Accordingly, polyphenol-rich berry extracts represent promising candidates for investigation as potential antifibrotic agents. The present study tested the hypothesis that polyphenol-rich berry extracts may attenuate the development of pulmonary fibrosis by modulating inflammatory responses and fibrotic tissue remodeling. Our previous studies have demonstrated the potential to attenuate the development of pulmonary fibrosis using polyphenolic extracts of grapes and bilberries [15,16].

The aim of this study was to evaluate the antifibrotic effects of concentrated polyphenolic extracts of bilberries (*V. uliginosum*), cranberries (*V. oxycoccos*), and lingonberries (*V. vitis-idaea*) in an experimental rat model of pulmonary fibrosis.

To test this hypothesis, we selected a model representing the late stage of pulmonary fibrosis. We used one of the most extensively validated experimental models of pulmonary fibrosis, the bleomycin-induced fibrosis model.

The bleomycin model is known to mimic many of the complex profibrotic responses typical of both idiopathic pulmonary fibrosis and interstitial lung diseases. Although this model does not reproduce the initiating cause of coal dust-associated lung disease, it reproduces many of the downstream inflammatory and fibrotic processes characteristic of progressive pulmonary fibrosis [17,18,19].

## 2. Materials and Methods

### 2.1. Polyphenol Extraction and HPLC Analyses

The extraction procedure and the compositional analysis of the polyphenolic extracts from *V. uliginosum*, *V. oxycoccos*, and *V. vitis-idaea* have been described previously [20].

Polyphenolic extracts from *V. uliginosum*, *V. oxycoccos* and *V. vitis-idaea* were prepared from wild berries during the summer–autumn season in the Surgut district of Khanty-Mansiysk County of the Tyumen region of the Russian Federation. Polyphenolic concentrates were obtained from berry skins by aqueous ethanol extraction. Extraction was performed using a 40% aqueous ethanol solution at a ratio of 1:5 (*w*/*v*), followed by concentration of the extract on a rotary evaporator to a dry matter content of 25%. The total polyphenol concentration was determined using a commercially available kit “Polyphenols Folin-Ciocalteu” (ENOLOGY line by BioSytems, Barcelona, Spain) according to the manufacturing guidelines.

Phenolic compounds were analyzed by high-performance liquid chromatography (HPLC) using an Agilent 1290 Infinity chromatograph (Agilent Technologies, Santa Clara, CA, USA), according to our previously published protocol, and the phenolic composition of these extracts has been described [21].

### 2.2. Animals

Male Wistar rats aged 3–4 months with a body weight of 290–320 g were used as laboratory animals. The animals were housed in cages at room temperature (22 ± 2 °C) under a 12-h light/dark cycle and a minimum relative humidity of 40%. They were fed a standard pelleted diet (V1534-300; ssniff Spezialdiäten GmbH, Soest, Germany), with food and water provided ad libitum.

Two weeks before the experiments, animals were randomly assigned to cages (3–4 rats per cage). The animals were used after a 14-day adaptation period. Cage allocation was maintained throughout the study to minimize handling-related stress.

### 2.3. Pulmonary Fibrosis Model Induced by Intratracheal Bleomycin Administration in Rats

Pulmonary fibrosis was induced by a single intratracheal (i.t.) administration of bleomycin (BLM) at a dose of 5 mg BLM/kg rat body weight.

Sixteen rats were randomly allocated to five experimental groups as follows:

**Group I (intact, *n* = 3):** Animals received a single intratracheal administration of 250 µL sterile saline. One week after i.t. saline administration, rats received drinking water via intragastric administration daily at a volume of 1.0 mL/rat for 14 days.

**Group II (control, *n* = 4):** Rats received a single intratracheal dose of BLM (5 mg/kg in 250 µL sterile saline). One week after BLM administration, animals received drinking water intragastrically at 1.0 mL/rat daily for 14 days.

**Group III (*V. uliginosum* extract, *n* = 3):** Rats received a single intratracheal dose of BLM (5 mg/kg in 250 µL sterile saline). One week later, they received *V. uliginosum* extract intragastrically at 1.0 mL/rat daily for 14 days. The polyphenol concentration in the *V. uliginosum* extract was 25.5 ± 0.9 mg/mL (Table 1). The polyphenol dose administered to the laboratory animals was 50 mg/kg rat body weight.

**Group IV (*V. oxycoccos* extract, *n* = 3):** Rats received a single intratracheal dose of BLM (5 mg/kg). One week later, they received *V. oxycoccos* extract intragastrically at 1.0 mL/rat daily for 14 days. The polyphenol concentration in the *V. oxycoccos* extract was 14.6 ± 0.9 mg/mL (Table 1). The polyphenol dose administered to the laboratory animals was 50 mg/kg rat body weight.

**Group V (*V. vitis-idaea* extract, *n* = 3):** Rats received a single intratracheal dose of BLM (5 mg/kg). One week later, they received *V. vitis-idaea* extract intragastrically at 1.0 mL/rat daily for 14 days. The polyphenol concentration in the *V. vitis-idaea* extract was 16.3 ± 1.7 mg/mL (Table 1). The polyphenol dose administered to the laboratory animals was 50 mg/kg rat body weight.

The extract administration schedule was selected based on the findings of the systematic review and meta-analysis by Qi Geng et al., which evaluated the efficacy and underlying mechanisms of flavonoids in animal models of bleomycin-induced pulmonary fibrosis [22]. This work indicates that flavonoids can mitigate pathological changes in the lungs with a prophylactic administration regimen. The authors suggest that flavonoids can alleviate pathological changes in the lungs. The beneficial effects of flavonoids on pulmonary fibrosis have been proposed to involve inhibition of inflammatory responses, restoration of oxidative and antioxidant homeostasis, and regulation of fibroblast proliferation, migration, and activation by transforming growth factor β1/mothers against the decapentaplegic homologue/AMP-activated protein kinase (TGF-β1/Smad3/AMPK), inhibitor kappa B alpha/nuclear factor-kappa B (IκBα/NF-κB), phosphatidylinositol 3-kinase (PI3K)/AKT, interleukin 6/signal transducer/activator of transcription 3 (IL6/STAT3), and nuclear factor erythroid 2-related factor 2/Kelch-like ECH-associated protein 1 (Nrf2-Keap1) pathways.

Euthanasia was performed 21 days after BLM administration and after the 14-day treatment period. After euthanasia by carbon dioxide inhalation using a rodent euthanasia unit (AE0904; RPC OpenScience Ltd., Krasnogorsk, Russian Federation), blood was collected from the unpaired posterior vena cava into EDTA-containing vacutainer tubes for subsequent hemorheological analysis. Following euthanasia, lung tissues were harvested and fixed in formalin for further histopathological analysis.

Blood viscosity was measured on a Brookfield DV2T rotational viscometer (AMETEK Brookfield, Middleboro, MA, USA) at various spindle speeds (2, 4, 6, 8, 12, 20, 40 and 60 rpm). A 700 µL aliquot of fresh blood collected from each laboratory animal immediately before viscosity measurement was placed on the temperature-controlled cone-and-plate measuring system of the viscometer maintained at 37 °C. The interval between blood collection and measurement was 5 min. Measurements were subsequently performed at progressively decreasing spindle speeds, beginning at 60 rpm and ending at 2 rpm.

### 2.4. Histological Examination

After removal of animals from the experiment, lung tissue sampling (including determination of optimal sampling site, specimen size, section orientation, and number of sections) was performed in accordance with the *Revised Guides for Organ Sampling and Trimming in Rats and Mice*, a joint publication of the RITA and NACAD groups (2003, Germany) [23].

To prevent postmortem collapse, the trachea was immediately bandaged, and the intact lungs were removed and immersed in 10% neutral buffered formalin (Sigma-Aldrich, St. Louis, MO, USA) for 24 h. Tissue samples were fixed in 10% formalin at a temperature of 4 °C for 24 h, rinsed with tap water and dehydrated using a series of alcohols of increasing concentration (70%, 90%, 95%, 100%). The tissue samples were then immersed in xylene and embedded in paraffin. Three consecutive 3-µm-thick histological sections were prepared from each paraffin block and mounted on glass slides. The slides were then dewaxed and stained.

Hematoxylin and eosin staining. The tissue sections were immersed in Mayer’s hematoxylin for a quarter of an hour, and then rinsed with water for 5 min. After that, the sections were counterstained with eosin for 1 min.

Masson trichrome staining. Bundles of type I collagen fibers were stained and distinguished from cellular components using Masson trichrome dye. A commercial kit (Trichrome dye (Masson)) was used for coloring with Masson trichrome Bio-Optica (Bio-Optica Milano S.p.A., Milan, Italy) according to the standard protocol. Collagen fibers were defined as dark blue fibers with black cores [24].

Histochemical staining of reticulin fibers was performed using Gomori’s silver impregnation method with the Reticulum staining kit (modified Gomori’s method; Bio-Optica (Italy)) according to the manufacturer’s standard protocol. Reticulin fibers were defined as black or dark brown fibers with gray nuclei [25].

For histochemical staining with orcein, a set of Orcein dyes (Bio-Optica, Italy) according to the manufacturer’s standard protocol was used for histological evaluation of elastic fibers. Elastic fibers were defined as dark brown fibers with gray nuclei.

### 2.5. Morphometric Examination

The morphometric analysis was carried out by two independent researchers blinded to group allocation and experimental intervention.

Three consecutive histological sections were analyzed for each animal. Histomorphological analysis was performed using a Zeiss AxioLab 4.0 light microscope (Zeiss, Oberkochen, Germany) equipped with a digital color micrograph system. Micrographs of representative histological sections were obtained using ImageJ software, version 1.53t (WS Rasband, NIH, Bethesda, MA, USA).

Hematoxylin and eosin staining was used to assess the overall histological architecture, interstitial pulmonary edema, and cellular infiltration. Edema was evaluated at ×40 magnification as the proportion of the histological section exhibiting edematous changes. Edema was scored as follows: 0, absent; 1, <10% of the section area; 2, 11–30%; and 3, >31%. Inflammatory lymphohistiocytic infiltration was assessed at ×40 magnification as the proportion of the histological section occupied by the inflammatory cellular infiltrate using the following scoring system: 0, absent; 1, <5% of the section area; 2, 6–15%; and 3, >16%. The numbers of multinucleated giant cells and mononuclear cells containing cytoplasmic inclusions were counted in 10 high-power fields at ×100 magnification, and the mean value was calculated for each animal.

Histopathological analysis of elastic fibers was performed in the central (perfusion) (respiratory bronchioles, arteries and veins) and peripheral (diffusion) respiratory compartments (microvessels, alveoli, respiratory bronchioles). The entire surface of each lung section was examined at ×10-, ×40-, and ×100-fold magnifications. In the central (perfusion) compartment, the predominant pattern of elastic fiber structure (thin filamentous fibers with a clear boundary/fibrous fibers with a blurred boundary) was determined. In the respiratory (diffusion) compartment, the predominant localization was evaluated: focal in the wall of thin alveolar septa/diffuse in the wall of thickened alveolar septa. The pattern of the native structure of elastic fibers was also evaluated considering structure (thin filamentous fibers with a clear boundary/fibrous fibers with a blurred boundary) and distribution (uniform/chaotic).

Morphometric analysis of type I collagen and type III collagen (reticulin) was used to assess pulmonary fibrosis. Pulmonary fibrosis is a histopathological pattern of lung damage in animals in an experimental study, which is a stress-induced (BLM) disorder of the histoarchitectonics of the respiratory tract with excessive deposition of extracellular connective tissue matrix (type I and III collagen) [26].

Type I collagen (mature collagen) is the most common collagen, which is a structural component of connective tissue and the predominant component of the interstitial membrane, forming the structural and mechanical basis (matrix) of bones, skin, tendons, cornea, walls of blood vessels and other connective tissues [27].

Type III collagen (other names: immature collagen, reticulin) is the second most abundant collagen expressed in early embryos and throughout embryogenesis. In adults, it is the main component of the extracellular matrix, distributed in the wall of large blood vessels and reticular fibers of hematopoiesis organs [28].

In the present study, the degree and stage of fibrosis were assessed as separate morphological parameters reflecting different characteristics of fibrotic lung injury.

The degree of fibrosis was also investigated. Morphometric analysis of collagen and reticulin assessed their presence in the respiratory compartment (microvessels, alveoli, respiratory bronchioles), and the amount of collagen/reticulin tissue was estimated in increments of 10% (10%, 20%, 30% and so on) on the image field. The entire surface of each histological section was examined at ×40 magnification. The degree of fibrosis was assessed semiquantitatively according to the percentage of the section area occupied by isolated or confluent fibrotic foci. Grade 0 (absent), <1%; Grade I (mild), 1–10%; Grade II (moderate), 11–30%; and Grade III (severe), >30%.

The stage of fibrosis was determined from the relative proportions of type I collagen and reticulin (type III collagen) within fibrotic foci. Histomorphometric assessment was performed at ×100 magnification. According to the predominance of mature collagen (type I collagen) or immature collagen (reticulin/type III collagen), fibrosis was classified into the following stages: FL0—absence of foci of pulmonary fibrosis; FL1—reticulin more than 80%, type I collagen less than 20%; FL2—reticulin more than 50%, type I collagen less than 50%; FL3—reticulin less than 50%, type I collagen more than 50%; FL4—reticulin less than 20%, type I collagen more than 80%.

### 2.6. Statistical Processing Methods

Statistical analyses were performed using IBM SPSS Statistics version 20.0 (IBM Corp., Armonk, NY, USA) and STATISTICA version 10.0. The Mann–Whitney U test was used to compare two independent groups. Multiple independent groups were compared using the Kruskal–Wallis test followed by Dunn’s post hoc test for multiple comparisons. Differences were considered statistically significant at *p* < 0.05.

### 2.7. Ethical Aspects

All research work with laboratory animals was carried out in accordance with generally accepted ethical standards for the treatment of animals, based on standard operating procedures that comply with the rules adopted by the European Convention for the Protection of Vertebrate Animals Used for Research and Other Scientific Purposes (Strasbourg, 1986) [29]. Permission was received from the Local Bioethics Commission of the Scientific Research Institute of Balneology and Medical Rehabilitation (protocol number No. 1-2024 dated 8 October 2024).

## 3. Results

### 3.1. Polyphenolic Composition of Bilberry (Vaccinium uliginosum), Cranberry (Vaccinium oxycoccos) and Lingonberry (Vaccinium vitis-idaea) Extracts

Table 1 summarizes the concentrations of the major polyphenolic components identified in the northern berry extracts.

The concentrations of individual phenolic compounds determined by HPLC are presented in Table 2.

### 3.2. Antifibrotic Effects of Polyphenolic Extracts from Bilberry (V. uliginosum), Cranberry (V. oxycoccos), Lingonberry (V. vitis-idaea) in a Rat Model of Bleomycin-Induced Pulmonary Fibrosis

#### 3.2.1. Macroscopic Examination of the Lungs

**Figure 1:**
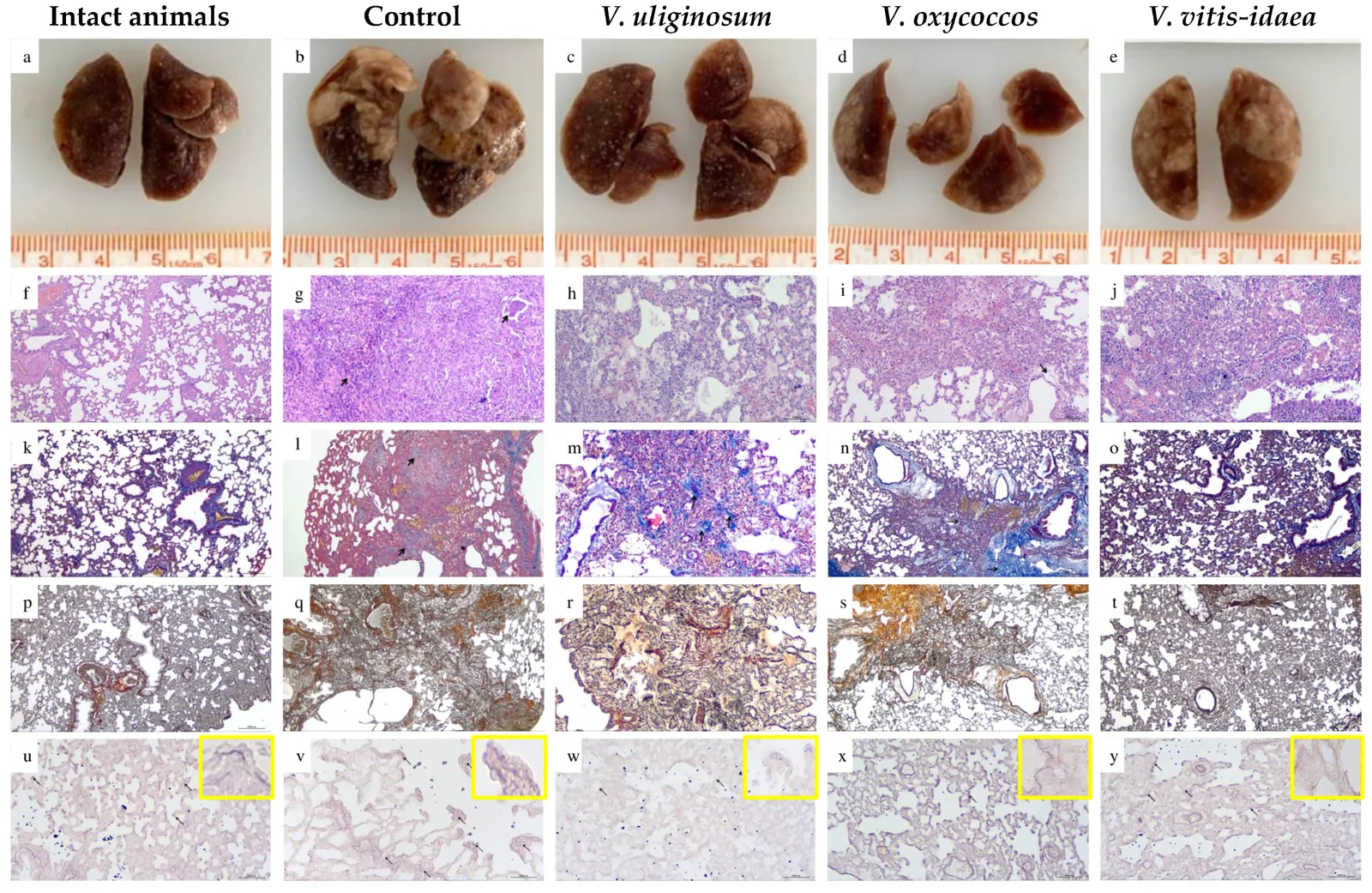
Representative gross lung images (**a**–**e**) and histological sections (**f**–**y**) from the experimental groups. **Intact animals**. (**a**) The visceral pleura was smooth, shiny, and uniformly grayish-red over the entire area (**f**). Lung histology was unremarkable: thin interalveolar septa without signs of edema and inflammatory infiltration. Hematoxylin and eosin, ×100, scale bar 500 μm. (**k**) Sparse collagen fibers were confined to the walls of large vessels and occasional peripheral regions of the respiratory compartment. Masson’s trichrome, ×100, scale bar 500 μm. (**p**) Thin filamentous fibers evenly distributed in the alveoli and respiratory bronchioles. Silvering by Gomory, ×100, scale bar 500 μm. (**u**) Sparse thin elastic fibers of dark red and brown color in the alveolar septa. Orcein, ×100, scale bar 500 μm. **Control**. (**b**) The lung surface exhibited multiple coalescing whitish lesions. (**g**) Multifocal lymphomacrophagal vasculitis and bronchiolitis, diffuse edema of the interalveolar septa, multi-row lymphohistiocytic infiltrate in the perivascular and peribronchial zones. Hematoxylin and eosin, ×100, scale bar 500 μm. (**l**) Histopathology of active prolonged fibrotic remodeling of lung tissue. Diffuse accumulations of thin filamentous structures in the alveoli and respiratory bronchioles. Masson’s trichrome, ×100, scale bar 500 μm. (**q**) The distribution of reticulin fibers is uniform, but with a diffuse character and discontinuity in the intraalveolar and intravascular spaces, an intermittent arrangement with uneven fiber thickness and multiple defibration sites on microfibers extending into the perivascular space. Silvering by Gomory, ×100, scale bar 500 μm. (**v**) Diffuse clusters of elastic fibers of various thicknesses, pronouncedly convoluted, located intermittently, with multiple areas of fibrillation in the thickness of the alveolar walls (arrows). Orcein, ×100, scale bar 500 μm. ***V. uliginosum*.** (**c**) The visceral pleura of the lungs is smooth and shiny, with dotted multifocal lesions interspersed inside fragments of normal areas of the lung, with the fusion of some foci. (**h**) Focal lymphomacrophagal vasculitis and bronchiolitis, with lymphohistiocytic infiltrates in perivascular and peribronchial areas. Hematoxylin and eosin, ×100, scale bar 500 μm. (**m**) Histopathology of focal inactive fibrotic remodeling of lung tissue. Filamentous collagen fibers located mainly in the area of the bronchiolo-alveolar junction. Masson’s trichrome, ×100, scale bar 500 μm. (**r**) Thin filamentous structures diffusely distributed in the alveoli and respiratory bronchioles. Silvering by Gomory, ×100, scale bar 500 μm. (**w**) Loose thin light brown elastin fibers. Orcein, ×100, scale bar 500 μm. ***V. oxycoccos***. (**d**) The surface of both lungs is reddish-gray, with dotted interspersed within fragments of normal areas of the lung, grayish-white, with the formation of slightly larger lesions. (**i**) Focal lymphohistiocytic infiltrate in the interalveolar septa. Hematoxylin and eosin, ×100, scale bar 500 μm. (**n**) Filamentous collagen fibers located mainly in the area of the bronchiolo-alveolar junction. Masson’s trichrome, ×100, scale bar 500 μm. (**s**) Uniform distribution of reticulin fibers. Silvering by Gomory, ×100, scale bar 500 μm. (**x**) Intermittent elastic fibers, represented by short, thin single filamentous structures of dark red and brown color. Orcein, ×100, scale bar 500 μm. ***Vaccinium vitis-idaea***. (**e**) Large merging lesions represented by dense white tissue. (**j**) Focal lymphocytic infiltration in the interstitium. Hematoxylin and eosin, ×100, scale bar 500 μm. (**o**) Histopathology of focal inactive fibrotic remodeling of lung tissue, ×100, scale bar 500 μm. (**t**) The distribution of reticulin fibers is uniform, but with diffuse character and discontinuity in the intraalveolar and intravascular spaces. Silvering by Gomory, ×100, scale bar 500 μm. (**y**) Single thin filamentous fibers of dark red and brown color (elastic fibers) in the walls of the alveoli. Orcein, ×100, scale bar 500 μm.

Representative photographs of the anterior lung surfaces from each experimental group are shown in Figure 1a–e. Macroscopic examination revealed marked differences in lung morphology among the experimental groups following bleomycin administration. Bleomycin-treated control animals exhibited multiple coalescing whitish lesions, whereas the extract-treated animals showed predominantly focal lesions interspersed with macroscopically preserved lung tissue.

In intact animals, the lungs exhibited normal gross morphology: the visceral pleura was smooth and glistening, and the lung surface was uniformly reddish-gray (Figure 1a). In the control group, multiple coalescing dense whitish foci were observed in both lungs (Figure 1b). In all extract-treated groups, the visceral pleura remained smooth and glistening, with multifocal lesions interspersed among macroscopically preserved reddish-gray lung tissue. No pleural retractions, surface deposits, or discrete pleural masses were observed (Figure 1c–e).

#### 3.2.2. Histological and Histomorphometric Examination of the Lungs

Histological and histomorphometric analyses were used to assess pulmonary inflammation, edema, and fibrosis in the experimental groups.

##### Histomorphometric Assessment of Inflammatory Cell Infiltration and Interstitial Edema

All intact animals exhibited normal lung histology, with thin interalveolar septa and no evidence of edema or inflammatory cell infiltration (Table 3, Figure 1f).

All bleomycin-treated control animals exhibited active chronic inflammation, characterized by severe diffuse polymorphonuclear leukocyte infiltration, moderate-to-severe plasma cell infiltration, abundant multinucleated giant cells, and marked interstitial edema (Table 3; Figure 1g). Mild inflammatory changes were not observed in this group.

Active chronic inflammation was not observed in any extract-treated animal. Two of the three *V. uliginosum*-treated animals exhibited moderate chronic nonspecific inflammation, whereas all *V. oxycoccos*- and *V. vitis-idaea*-treated animals and the remaining *V. uliginosum*-treated animal showed mild inactive inflammation. Inflammatory infiltrates were predominantly microfocal and consisted of polymorphonuclear leukocytes and plasma cells. Plasma cell infiltration was moderate in the two *V. uliginosum*-treated animals with moderate inflammation and mild in the remaining extract-treated animals. Multinucleated giant cells were absent from all extract-treated groups, whereas macrophages with cytoplasmic inclusions were present in all extract-treated animals. Interstitial edema was moderate in the *V. uliginosum* group and mild and focal in the *V. oxycoccos* and *V. vitis-idaea* groups (Table 3; Figure 1h–j).

##### Histomorphometric Assessment of the Severity of Pulmonary Fibrosis in the Experimental Groups

**Figure 2:**
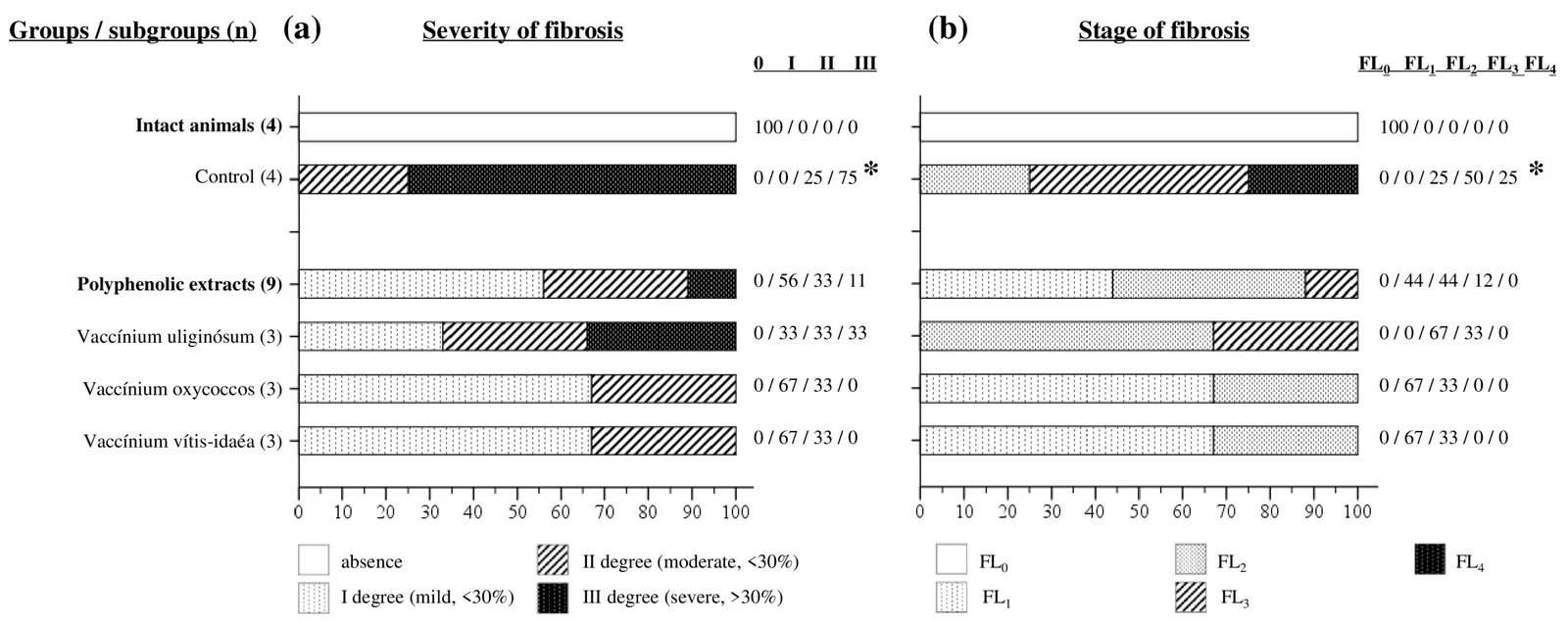
Histomorphometric assessment of pulmonary fibrosis severity and stage in intact rats and rats with bleomycin-induced pulmonary fibrosis: (**a**) severity of pulmonary fibrosis; (**b**) stage of pulmonary fibrosis (FL0–FL4). The control group exhibited a diffuse pattern of lung injury with predominantly severe fibrosis and advanced fibrosis stages. In the berry extract-treated groups, fibrotic remodeling was predominantly focal or microfocal. * *p* < 0.05 for the control group versus the intact group and versus each of the *V. uliginosum, V. oxycoccos*, and *V. vitis-idaea* groups. FL, histopathological fibrosis stage (FL0–FL4) as defined in the Section 2.

In the intact group, no histoarchitectural abnormalities associated with fibrosis were detected. Sparse thin filamentous type I collagen fibers occupied less than 5% of the examined area, while thin reticulin fibers were evenly distributed throughout the alveoli and respiratory bronchioles (Table 4; Figure 2a).

In the control group, the mean area of fibrotic lung involvement was 59.4 ± 11.8%, corresponding to a diffuse pattern of injury. Severe fibrosis was observed in three animals (75%), whereas moderate fibrosis was observed in one animal (25%). No animals exhibited mild fibrosis or an absence of fibrosis (Table 4; Figure 2a).

Across all berry extract-treated groups, the mean area of fibrotic lung involvement was less than 25%. Mild fibrosis was detected in five of the nine extract-treated animals (56%): one of three animals in the *V. uliginosum* group (33%), two of three in the *V. oxycoccos* group (67%), and two of three in the *V. vitis-idaea* group (67%). Moderate fibrosis was detected in three of the nine extract-treated animals (33%), including one of three animals in each of the *V. uliginosum, V. oxycoccos,* and *V. vitis-idaea* groups (33% per group). Severe fibrosis was observed in only one of the nine extract-treated animals (11%), corresponding to one of three animals in the *V. uliginosum* group (33%) (Table 4; Figure 2a).

##### Histomorphometric Assessment of Pulmonary Fibrosis Stage and Fibrous Tissue Composition

In the control group, the histological pattern of fibrous tissue was characterized by a relative predominance of mature type I collagen within pulmonary fibrotic foci in three of four animals (75%). The fibrosis pattern corresponded to stage FL3 in two animals (50%) and to stage FL4 in one animal (25%). A focal distribution of mature type I collagen within a diffuse background of immature resorbable collagen fibers, corresponding to stage FL2, was observed in only one animal (25%). No control animals exhibited stage FL1, characterized by a minimal amount of mature stable type I collagen, or stage FL0, indicating the absence of mature stable type I collagen (Table 4; Figure 2b).

Across the berry extract-treated groups, the histological pattern of fibrous tissue was characterized by a predominance of immature resorbable type III collagen fibers (reticulin) in eight of nine animals (88%). In 4 cases (44%), the fibrous tissue pattern with a pronounced predominance of reticulin (FL1) prevailed in subgroups with cranberry *V. oxycoccos* (67%) and lingonberry *V. vitis-idaea* (67%). Stage FL2, characterized by a moderate predominance of reticulin, was observed in four of the nine extract-treated animals (44%): two of three animals in the *V. uliginosum* group (67%), one of three in the *V. oxycoccos* group (33%), and one of three in the *V. vitis-idaea* group (33%) (Table 4; Figure 2b).

A fibrous tissue pattern with a predominance of mature nonresorbable collagen was observed only in the *V. uliginosum* group, in one of three animals (33%). In this animal, the fibrosis pattern corresponded to stage FL3, characterized by a moderate predominance of mature nonresorbable type I collagen fibers (Table 4; Figure 2b). Stage FL4, characterized by a pronounced predominance of mature collagen, was not observed in any extract-treated animal, in contrast to the control group.

In all intact animals, orcein staining revealed thin, well-defined elastin fibers that were evenly distributed throughout the lung tissue (Figure 1u; Table 4).

In the control group, early elastolysis was observed in the peripheral respiratory compartment in all four animals (100%). The elastic fibers were fragmented, sparse, unevenly distributed, and thickened. In two animals (50%), elastic fibers were diffusely distributed within the thickened alveolar septa (Figure 1v; Table 4).

Across all berry extract-treated groups, elastic fibers were focally distributed within the walls of thin alveolar septa in all nine animals (100%). In 1 (33%) case, diffuse fibrous elastin fibers were observed in the *V. uliginosum* and *V. vitis-idaea* groups (Figure 1w–y; Table 4). Diffusely distributed fibrous elastin structures were observed in one of three animals (33%) in the *V. uliginosum* group and in one of three animals (33%) in the *V. vitis-idaea* group (Figure 1w–y; Table 4).

### 3.3. Effects of Polyphenolic Extracts from V. uliginosum, V. oxycoccos, and V. vitis-idaea on Hemorheological Parameters

Blood viscosity was measured in male rats using a Brookfield DV2T rotational viscometer over a spindle speed range of 2–60 rpm. Measurements obtained across different spindle speeds provide information on hemorheological behavior under varying shear conditions, which is commonly interpreted in relation to erythrocyte aggregation at low shear rates and erythrocyte deformability at high shear rates [30].

**Figure 3:**
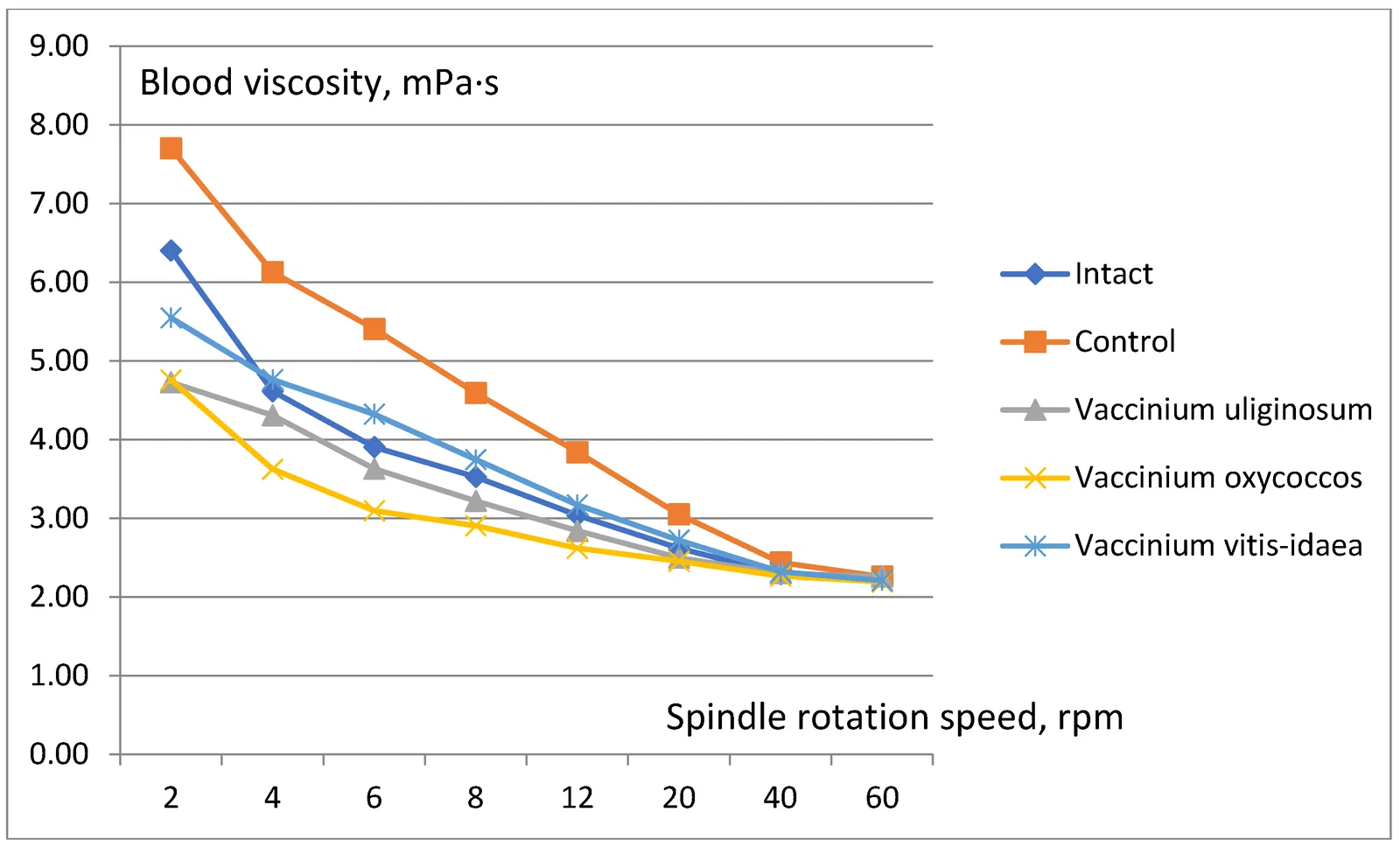
Hemorheological activity of plant polyphenols of bilberry (*V. uliginosum*), cranberry (*V. oxycoccos*) and lingonberry (*V. vitis-idaea*).

Intratracheal administration of bleomycin was associated with increased blood viscosity in rats with bleomycin-induced pulmonary fibrosis (Table 5). As shown in Table 5 and Figure 3, blood viscosity was higher in the control group than in the intact group at almost all spindle speeds.

Previous studies have shown that bleomycin administration may be accompanied by systemic vascular and hematological changes in addition to pulmonary injury, including endothelial activation and alterations in circulating blood cells [31,32]. Although these parameters were not evaluated in the present study, such changes may contribute to the increased blood viscosity observed in the bleomycin-treated animals.

Course administration of extracts of bilberry *V. uliginosum*, cranberry *V. oxycoccos* and lingonberry *V. vitis-idaea* in an experimental model of pulmonary fibrosis contributed to a decrease in blood viscosity. Treatment resulted in a decrease in blood viscosity, mainly at low and medium shear rates, indicating reduced erythrocyte aggregation and improved deformability.

## 4. Discussion

The present study evaluated the antifibrotic effects of polyphenolic extracts from *Vaccinium uliginosum*, *Vaccinium oxycoccos*, and *Vaccinium vitis-idaea* in a rat model of bleomycin-induced pulmonary fibrosis.

Histopathological analysis demonstrated that treatment with berry extracts was associated with significantly less severe pulmonary fibrosis than that in the untreated control group (*p* < 0.05). In addition to reducing the overall extent of fibrosis, the extract-treated groups exhibited a predominance of immature type III collagen (reticulin), whereas the control group showed predominantly mature type I collagen within diffuse fibrotic lesions. These findings are consistent with previous experimental studies reporting antifibrotic effects of biologically active compounds in bleomycin-induced pulmonary fibrosis [15,16,33,34].

Berry extract treatment was also associated with significantly less inflammatory cell infiltration than that observed in untreated animals. Histologically, the extract-treated groups demonstrated reduced granulocyte and plasma cell infiltration, whereas the control group exhibited extensive inflammatory infiltrates accompanied by multinucleated giant cells. Collectively, these findings suggest that berry extracts may exert local anti-inflammatory effects that contribute to attenuation of pulmonary fibrosis.

Numerous macrophages containing abundant intracytoplasmic inclusions were observed within areas of immature fibrous tissue in the extract-treated groups, where they displayed a foam cell-like morphology. In contrast, the untreated control group contained relatively few such macrophages but exhibited a greater abundance of multinucleated giant cells, indicating a distinct inflammatory cell profile. Although macrophage function was not directly investigated, the increased abundance of morphologically active macrophages in the treated groups may be consistent with enhanced clearance of tissue debris and resolution of local inflammation. However, the cellular and molecular mechanisms underlying these observations remain to be established.

No histological evidence of local immunosuppression was identified in the berry extract-treated groups. Previous studies have shown that pharmacological immunosuppression may be associated with increased synthesis of immature type III collagen accompanied by a relative reduction in mature collagen [35]. In contrast, the present study demonstrated an overall reduction in fibrotic tissue together with decreased inflammatory cell infiltration, findings that are more consistent with local immunomodulation than nonspecific immunosuppression. Nevertheless, this interpretation should be considered tentative pending mechanistic investigation.

Rats treated with the *V. uliginosum* extract demonstrated more extensive multifocal perivascular edema than animals receiving *V. oxycoccos* or *V. vitis-idaea*, in which edema was limited to focal or microfocal areas. Although the biological significance of this finding remains uncertain, it may reflect differences in the degree of residual acute interstitial injury among the treatment groups.

Among the three berry extracts evaluated, *V. oxycoccos* and *V. vitis-idaea* demonstrated the most favorable qualitative histopathological profile, characterized by minimal inflammatory cell infiltration and a predominance of immature type III collagen. Although quantitative differences in fibrosis severity among the extract-treated groups did not reach statistical significance, these qualitative findings may indicate differences in extracellular matrix remodeling that warrant further investigation.

Morphometric analysis further demonstrated better preservation of elastic fibers in the berry extract-treated groups than in untreated controls. Histologically, elastic fibers remained thinner and more clearly delineated within relatively preserved alveolar septa, whereas the control group exhibited diffuse disruption and architectural distortion of elastic fibers within markedly thickened septa. These findings further support a protective effect of berry extracts on lung tissue architecture.

To place these observations into context, Li et al. demonstrated, using both a bleomycin-induced rat model and cultured pulmonary fibroblasts, that blueberry-derived polyphenols attenuated pulmonary fibrosis through suppression of TGF-β1-associated profibrotic signaling [36]. The authors also reported inhibition of epithelial–mesenchymal transition (EMT), reduced fibroblast migration, and increased E-cadherin expression, suggesting multiple mechanisms by which polyphenols may limit fibrogenic remodeling.

Although these findings provide a plausible biological framework for the present observations, the current study did not investigate molecular signaling pathways. Therefore, any involvement of EMT, TGF-β1/Smad signaling, or related mechanisms in the antifibrotic effects of *V. uliginosum*, *V. oxycoccos*, and *V. vitis-idaea* remains speculative and requires direct experimental confirmation.

The present findings indicate that polyphenolic extracts from *V. uliginosum*, *V. oxycoccos*, and *V. vitis-idaea* possess antifibrotic potential in this experimental model, likely through combined modulation of inflammatory responses and extracellular matrix remodeling. However, their efficacy and safety in human fibrotic lung diseases, including occupational lung disorders such as coal mine dust lung disease, remain unknown and should not be inferred from the present preclinical data.

Further studies are warranted to elucidate the molecular mechanisms underlying these effects, determine the contribution of individual polyphenolic compounds, evaluate bioavailability and metabolic transformation, and assess long-term efficacy and safety. Such investigations will be essential for determining the translational potential of berry-derived polyphenols as candidate antifibrotic agents.

## Figures and Tables

**Table 1:** Total concentrations of the major polyphenolic components in concentrates from *V. uliginosum*, *V. oxycoccos* and *V. vitis-idaea*.

Berry Extract	Phenols mg GAE/mL	Flavonoids μmol CE/mL	Anthocyanins mg C3G/mL
*Vaccínium uliginósum*	25.5 ± 0.9	10.2 ± 0.2	19.7 ± 0.8
*V. oxycoccos*	14.6 ± 0.9	8.3 ± 0.3	2.1± 0.5
*Vaccínium vítis-idaéa*	16.3 ± 1.7	9.9 ± 1.2	4.0 ± 0.9

Data are presented as the mean ± standard deviation of five independent analytical measurements (*n* = 5).

**Table 2:** Concentrations of individual phenolic compounds in berry extracts (mg/L) in concentrates from *V. uliginosum*, *V. oxycoccos* and *V. vitis-idaea*.

Phenolic Compound	*Vaccínium uliginósum*	*Vaccinium oxycoccos*	*Vaccínium vítis-idaéa*
Gallic acid	3.66 ± 0.06	4.18 ± 1.06	2.11 ± 0.86
Protocatechuic acid	106.8 ± 10.6	5.4 ± 1.7	12.6 ± 1.4
Procyanidin (total)	345.8 ± 10.2	53.7 ± 9.0	48.6 ± 11.3
Procyanidin A	61.4 ± 8.8	17.2 ± 3.6	23.8 ± 5.2
Procyanidin B1	22.6 ± 1.9	ND	ND
Procyanidin B2	118.3 ± 13.4	18.4 ± 0.9	13.6 ± 4.4
Procyanidin C	94.1 ± 10.6	8.4 ± 1.1	ND
3-*p*-Coumaroylquinic acid	206.5 ± 24.9	30.3 ± 2.8	95.1 ± 11.7
(+)-Catechin	ND	113.5 ± 9.8	438.4 ± 31.4
Chlorogenic acid (5-Caffeoylquinic acid)	88.0 ± 9.7	533.3 ± 63.7	240.5 ± 16.0
trans-Caffeic acid	42.5 ± 3.8	4.3 ± 0.6	13.7 ± 0.8
(−)-Epicatechin	106.3 ± 13.7	182.0 ± 12.6	176.2 ± 9.8
trans-p-Coumaric acid	5.1 ± 0.6	83.7 ± 9.1	139.8 ± 19.4
Myricetin-glycoside	ND	204.4 ± 39.5	ND
trans-Ferulic acid	ND	13.3 ± 0.8	51.8 ± 8.8
Quercetin-3-glucuronide	23.8 ± 1.6	61.9 ± 9.8	12.6 ± 4.2
Quercetin-3-galactoside	8.2 ± 0.9	80.5 ± 9.1	41.7 ± 6.7
Quercetin 3-glucoside	118.2 ± 8.5	13.5 ± 2.2	26.4 ± 7.0
Pelargonidin	17.7 ± 2.4	ND	ND
trans-Resveratrol	18.8 ± 3.5	3.7 ± 0.9	ND
Quercetin 3-xyloside	9.4 ± 1.3	8.8 ± 0.7	11.9 ± 2.4
Quercetin 3-arabinopyranoside	ND	ND	22.4 ± 2.8
Quercetin 3-arabinofuranoside	ND	ND	20.8 ± 3.1
Kaempferol 3-glucoside	18.4 ± 3.1	ND	ND
Quercetin-3-rhamnoside	40.6 ± 2.2	40.3 ± 8.1	91.3 ± 11.7
Myricetin	16.7 ± 4.1	9.2 ± 1.4	ND
Kaempferol	ND	ND	17.0 ± 2.5
Hydroxybenzoic acids	115.7 ± 8.4	217.5 ± 28.3	170.0 ± 17.8
Hydroxycinnamic acids	133.9 ± 49.9	116.9 ± 27.4	204.7 ± 18.6
Flavonols	730.0 ± 58.2	1524.0 ± 168.3	1958.3 ± 104.7
Flavan-3-ols	1007.5 ± 162.0	1539.5 ± 206.1	2117.0 ± 180.4
Delphinidin	725.4± 55.2	10.3 ± 0.7	ND
Cyanidin	608.6 ± 113.7	68.5 ± 13.6	127.0 ± 30.9
Peonidin	804.5 ± 93.3	50.3 ± 7.4	11.7 ± 0.7
Malvidin	117.8 ± 7.4	8.4 ± 1.7	13.6 ± 0.9

Data are presented as the mean ± standard deviation of five independent analytical measurements (n = 5). ND—not detected.

**Table 3:** Histomorphometric characteristics of interstitial edema and cellular infiltration.

Histo- Morphometric Parameter	Intact Animals (n = 3) (M ± SD)	Control (n = 4) (M ± SD)	*V. uliginosum* (n = 3) (M ± SD)	*V. oxycoccos* (n = 3) (M ± SD)	*V. vitis-idaea* (n = 3) (M ± SD)
Edema *	-	3.0 ± 0.0	2.3 ± 0.6	1.0 ± 0.5	1.0 ± 1.0
Polymorphonuclear leukocytes **	-	3.0 ± 0.0	1.0 ± 0.0	1.0 ± 0.0	1.0 ± 0.0
Plasma cells **	-	2.3 ± 0.9	1.7 ± 0.6	1.0 ± 0.0	1.0 ± 0.0
Multinucleated giant cells ***	-	3.0 ± 0.0	0.0 ± 0.0	0.0 ± 0.0	0.0 ± 0.0
Macrophages with cytoplasmic inclusions (foam cells) ***	-	1.3 ± 0.5	2.7 ± 0.6	3.0 ± 0.0	3.0 ± 0.0

* Edema score: 0. absent; 1, involving <10% of the examined area; 2, involving 11–30%; and 3, involving >30%. ** Inflammatory cell infiltration score: 0, <1% of the examined area, with no active inflammation; 1, <10%, with microfocal mild inflammation; 2, 10–30%, with multifocal or confluent moderate inflammation; and 3, >30%, with diffuse severe inflammation. *** Multinucleated giant cells and macrophages with cytoplasmic inclusions were scored separately as follows: 0, absent; 1, 1–5 cells; 2, 6–10 cells; and 3, >10 cells.

**Table 4:** Histomorphometric characteristics of the stage and degree of fibrosis.

Criteria	Intact Animals (*n* = 3)	Control (*n* = 4)	*V. uliginosum* (*n* = 3)	*V. oxycoccos* (*n* = 3)	*V. vitis-idaea* (*n* = 3)
**Degree of fibrosis**					
<1% (grade 0-none)	100% (3/3)	-	-	-	-
<10% (grade I-mild)	-	-	33% (1/3)	67% (2/3)	67% (2/3)
<30% (grade II-moderate)	-	25% (1/4)	33% (1/3)	33% (1/3)	33% (1/3)
>30% (grade III-severe)	-	75% (3/4)	33% (1/3)	-	-
M ± SD	2.6 ± 0.3	59.4 ± 11.8	16.2 ± 8.8	11.2 ± 4.5	13.2 ± 5.9
**Stage of fibrosis**					
FL0 (N/%)	100% (3/3)	-	-	-	-
FL1 (N/%)	-	-	-	67% (2/3)	67% (2/3)
FL2 (N/%)	-	25% (1/4)	67% (2/3)	33% (1/3)	33% (1/3)
FL3 (N/%)	-	50% (2/4)	33% (1/3)	-	-
FL4 (N/%)	-	25% (1/4)	-	-	-
M ± SD (collagen type III/I)	-	40.4 ± 11/50.4 ± 15	63.3 ± 16/46.4 ± 19	76.3 ± 11/30.4 ± 13	69.3 ± 17/35.4 ± 16

Data are presented as percentage (N/n), where N is the number of animals with the corresponding severity or stage of fibrosis and n is the total number of animals in the group.

**Table 5:** Blood viscosity indices in rats with an experimental model of pulmonary fibrosis.

The Study Group	Blood Viscosity (mPa s) at Different Spindle Speeds, Revolutions per Minute							
	2	4	6	8	12	20	40	60
Intact animals, n = 3	6.40 ± 0.11	4.61 ± 0.15	3.90 ± 0.11	3.52 ± 0.08	3.04 ± 0.08	2.62 ± 0.06	2.29 ± 0.10	2.25 ± 0.10
Control, n = 4	7.70 ± 0.46 *p*1 = 0.0499	6.13 ± 0.33 *p*1 = 0.0081	5.41 ± 0.16 *p*1 = 0.0003	4.59 ± 0.04 *p*1 = 0.00001	3.84 ± 0.23 *p*1 = 0.0236	3.05 ± 0.17 *p*1 = 0.0763	2.44 ± 0.06 *p*1 = 0.2959	2.26 ± 0.06 *p*1 = 0.9798
*Vaccinium uliginosum*, n = 3	4.73 ± 0.38 *p*2 = 0.0034	4.31 ± 0.29 *p*2 = 0.0083	3.63 ± 0.23 *p*2 = 0.0008	3.22 ± 0.21 *p*2 = 0.0008	2.84 ± 0.16 *p*2 = 0.0176	2.50 ± 0.12 *p*2 = 0.0551	2.30 ± 0.08 *p*2 = 0.2795	2.26 ± 0.09 *p*2 = 0.9843
*Vaccinium oxycoccos*, n = 3	4.76 ± 0.05 *p*2 = 0.0009	3.63 ± 0.04 *p*2 = 0.0003	3.10 ± 0.02 *p*2 = 0.00001	2.91 ± 0.04 *p*2 = 3 × 10^−8^	2.62 ± 0.07 *p*2 = 0.0031	2.46 ± 0.11 *p*2 = 0.0372	2.26 ± 0.04 *p*2 = 0.0806	2.19 ± 0.03 *p*2 = 0.4272
*Vaccinium vitis-idaea*, n = 3	5.55 ± 0.71 *p*2 = 0.0606	4.76 ± 0.56 *p*2 = 0.1088	4.32 ± 0.64 *p*2 = 0.1912	3.75 ± 0.41 *p*2 = 0.1103	3.17 ± 0.33 *p*2 = 0.1840	2.72 ± 0.19 *p*2 = 0.2927	2.32 ± 0.06 *p*2 = 0.2766	2.21 ± 0.03 *p*2 = 0.5617

Note: n is the number of animals in the group; *p* is the significance level.; *p*1 < 0.05–statistically significant differences compared with the values in the intact group; *p*2 < 0.05–statistically significant differences compared with the corresponding values in the control group.

## Data Availability

The original contributions presented in this study are included in the article. Further inquiries can be directed to the corresponding authors.

## Abbreviations

The following abbreviations are used in this manuscript:

CMDLD	coal mine dust lung disease
EMT	epithelial–mesenchymal transition
BLM	bleomycin
PF	pulmonary fibrosis
rpm	revolutions per minute
